# PD-L1 expression on monocytes marks active systemic lupus erythematosus in patients without nephritis

**DOI:** 10.1186/1546-0096-10-S1-A2

**Published:** 2012-07-13

**Authors:** Anne M Stevens, Jing-Ni Ou

**Affiliations:** 1 Seattle Children's Research Institute, Seattle, WA, USA; 2 University of Washington, Seattle, WA, USA

## Purpose

Programmed death ligand 1 (PD-L1) plays an important role in controlling autoreactive and follicular T helper lymphocytes, which can induce autoantibody production. Properly expressed PD-L1 thus prevents autoimmune disease. Induced by inflammatory cytokines, PD-L1 is elevated on monocytes from patients with infections and rheumatoid arthritis, but is poorly expressed on monocytes from SLE patients with active disease. Our goal was to define the mechanism for PD-L1 dysregulation in pediatric patients with SLE, and to clinically characterize those patients with loss of PD-L1.

## Methods

Serial samples were obtained from 26 pediatric patients with SLE (20 samples during active disease, SELENA-SLEDAI score >4, and 37 during remission) and 28 age-matched controls. PD-L1 expression on CD11c^+^ CD14^high^ monocytes was assayed in cultured peripheral blood cells by flow cytometry. Cytokines in culture supernatants were assayed by multiplex ELISA.

## Results

PD-L1 expression on healthy control monocytes was 2.7-fold higher than SLE patients with active disease (mean induction 28.7, SD 17.7, versus 10.5, SD 11.4, p<0.005). Overall, SLE patients in remission expressed normal levels of PD-L1 on monocytes (mean 24.2, SD 16.5, p<0.0005 versus active SLE). PD-L1 surface expression was induced on a mean of 80% of healthy monocytes, compared to patients with active SLE (mean 52%, p=0.00003). ROC curves demonstrated the ability of PD-L1 to distinguish between active and inactive disease (AUC 0.75) and between active SLE and healthy controls (AUC 0.79). The effect was most pronounced in patients *without* renal disease; patients with any history of renal disease did not restore monocyte PD-L1. Patients in remission with previous renal disease had a50% of the PD-L1 expression of those in remission who never had renal disease (mean 14.0 vs. 29.1, p=0.02). Non-renal patients restored PD-L1 fully during remission, and disease activity correlated with PDL1 expression (Spearman correlation r= -0.38, P=0.02). PD-L1 expression on active SLE monocytes could be restored by healthy cells producing IL-10 and/or TNF-α. Conversely, PD-L1 expression on healthy monocytes was suppressed by TGF-β, which SLE cells over-expressed.

## Conclusion

During an inflammatory response patients with SLE are lacking a key step to limit autoreactive lymphocyte activity: the local, cytokine-mediated induction of PD-L1 expression on antigen presenting cells. Moreover, therapeutic agents that inhibit TNF-α may induce autoantibodies in part by preventing PD-L1 induction, thereby impeding an important mechanism of T and B lymphocyte regulation. Patients with a persistent defect in PD-L1 expression may be prone to more severe disease, leading to glomerulonephritis, whereas patients who can express PD-L1 after immunosuppressive therapy can resolve autoantibody production, preventing the progression toward renal disease. Infection and other autoimmune diseases may potentially be distinguished from active non-renal SLE on the basis of monocyte PD-L1 expression.

## Disclosure

Anne M. Stevens: None; Jing-Ni Ou: None.

